# Real-Time PCR to Phenotype Resistance to the Citrus Nematode *Tylenchulus semipenetrans* Cobb.

**DOI:** 10.3390/plants12132543

**Published:** 2023-07-04

**Authors:** Marta Ruiz, Annie Du Vo, J. Ole Becker, Mikeal L. Roose

**Affiliations:** 1 Department of Botany and Plant Sciences, University of California, Riverside, CA 92521, USA; 2 Department of Nematology, University of California, Riverside, CA 92521, USA; obecker@ucr.edu

**Keywords:** plant parasitic, rootstock, breeding, Poncirus, trifoliate, extraction, quantification, qPCR, screening, pest

## Abstract

As pest management options, such as nematicides, become more restrictive, developing rootstocks resistant to the citrus nematode is fundamental for citrus production. This study provides an updated methodology to screen for citrus nematode resistance in rootstock-breeding programs. We developed a novel method to extract female citrus nematodes from roots that is suitable for molecular work and a real-time-PCR-based nematode quantification method for *Tylenchulus semipenetrans*. These procedures allow scaling up screening to high-throughput workflows, increasing the chances of finding rootstock candidates that combine all the desired traits. Our results contribute to the growing literature supporting quantification of nematodes with molecular methods.

## 1. Introduction

Rootstocks are a fundamental pillar of citrus production. Root traits are closely linked to many aspects of tree performance, such as fruit quality, adaptation to the environment, and resistance to diseases, among others. Thus, rootstock improvement is a key component in citrus-breeding programs as it provides new rootstock types that combine the beneficial traits required to address the current and emerging limitations in production. The citrus nematode, *Tylenchulus semipenetrans* Cobb. (CN), is the causal agent of slow decline (SD) disease, which is present in all citrus-growing areas in the world. CN spread through the exchange of nursery stocks in the past and has persisted, as new healthy trees are planted in infested grove sites or infested seedlings and management practices bring it to new planting sites [1,2]. SD disease affects newly planted young trees by hampering their establishment, while older infested trees usually show poor performance, low yield, and low fruit quality. CN slowly drains carbon from the tree. Initially, the overall effect might be mild, but with enough time, it becomes significant. The parasitized root system develops poorly and typically has shorter, thicker roots that look dirty as soil particles adhere to the nematode egg masses. SD disease has no remedy or cure and makes the tree more susceptible to other diseases. SD disease is prevented by using land, stocks, and water that are free of *T. semipenetrans* and is usually managed with nematicide applications [1]. Damage thresholds require local evaluation as they are influenced by the nematode reproductive rate, rootstock susceptibility, climate, soil properties, management practices, and interaction with other pathogens or microbial antagonists [3,4,5]. However, nematicides only reduce populations temporarily, and their effects on the tree and production are limited and delayed. The development of sensitive detection methods based on PCR has aided CN management [6,7]. However, the most efficient way to tackle SD disease is to use rootstocks that are resistant to CN. As new rootstock types are developed, it is crucial to screen for resistance among progenies, and some breeders consider this trait essential for final selection.

The first-stage juvenile develops and molts inside an egg, and the second-stage juvenile (J2) hatches soon after. About one-fourth of the population develops into males. They stay vermiform and mobile throughout life and do not feed or enter the roots. The remaining J2 individuals represent the infective and persistent stage that feeds in an ectoparasitic manner. However, only a small percentage eventually enters the feeder roots and moves into the cortical endodermis. After the fourth molt, the young female penetrates further into the cortex. The sedentary adult citrus nematode induces several nurse cells that serve as permanent feeding sites for the rest of her life. The body elongates, and the posterior end swells and expands from the root surface, ending with a fingerlike protuberance. The females lay about 100 eggs. These are enclosed in a gelatinous matrix that protects the progeny from environmental and biological threats. Under ideal moisture conditions, environmental temperatures between 20 °C and 30 °C determine the developmental time from egg to egg. The largest populations of citrus nematodes occur in the spring and autumn following the citrus root flushes. Populations tend to peak when resources are stable for a few weeks [8]. The abundance of feeder females in the roots is the best indicator of seasonal activity, and it is used to determine damaging thresholds in SD disease management [9]. Resistant rootstocks are those able to develop defense responses against female penetration into the root cortex and hamper the establishment of a permanent feeding site that inhibits reproduction. The defense response may include the production of anti-nematode enzymes, compounds such as reactive oxygen species and hormones, the hypersensitive-response-mediated death of surrounding cells, and cell wall reinforcement at penetration sites [10,11,12].

CN is one of the most highly specialized parasites among plant-feeding nematodes as it can only infest specific hosts. There are three biotypes of CN, known as ‘Citrus’, ‘Poncirus’, and ‘Mediterranean’. These biotypes differ in their host range and distribution. The ‘Citrus’ and ‘Mediterranean’ types are most common in California, Japan, Italy, and Spain [13]; they can infest all *Citrus* species, but they cannot invade the citrus relative *Poncirus trifoliata* Raf, which is considered the standard of resistance for citrus breeding. The ‘Poncirus’ type has been rarely reported, but it is known to be present in California, Japan, Israel, and Spain [14,15,16]). This biotype can infest both *Citrus* species and *P. trifoliata*. Therefore, it is a resistance-breaking population and is considered an emerging challenge.

The citrus relative *P. trifoliata*, widely used as a parent for rootstock breeding, is the only germplasm source of CN resistance incorporated into commercial citrus rootstocks [17]. Some *Citrus* × *Poncirus* hybrids are susceptible, while others are resistant to the ‘Citrus’ and ‘Mediterranean’ CN types. Genetic studies suggest that there are probably one or two major genes located at the *Tyr1* locus that are involved in resistance and some others of minor effect [18,19,20], which creates a continuous phenotypic distribution, with moderately sensitive types in between the more resistant and the more susceptible types. Citrus-breeding programs based on sexual hybridization involve logistical and time constraints, such as juvenility, plant size, high heterozygosity, and nucellar embryony or quantitative traits, that complicate the identification of required characters and the selection of individuals that meet all the requirements. New genomic information and the development of fast, early, and efficient screenings for phenotyping facilitate the development of new rootstocks that combine all the desired traits. With the current threat of Huanglongbing disease, which significantly impacts root function, plant nutrition, and fruit quality, addressing the detrimental effects of CN on citrus cultivation is critically important.

A previous study by Niles et al. [21] reported that female CN abundance in the roots is the best indicator of rootstock resistance to CN and proposed using *P. trifoliata* as a standard of resistance to perform screenings based on a one-to-one comparison. The study also showed that the test is more accurate and efficient when using small containers rather than bigger pots. Our study aims to provide an updated methodology based on previous research suitable for high-throughput evaluations. For that purpose, we designed a novel technique to extract CN females based on sand abrasion and a qPCR method to quantify the abundance of CN females infesting citrus roots.

## 2. Results

### 2.1. qPCR Assay

#### 2.1.1. Specificity

**Figure 1:**
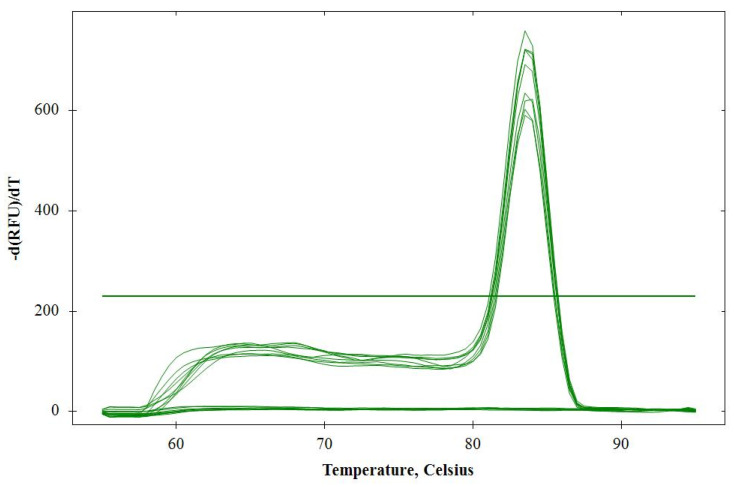
Melting curve profile of *Tylenchulus semipenetrans* amplicons with the melting temperature at 83.5 °C. No amplification was observed in control reactions without *T. semipenetrans* DNA.

The primer pair selected (Table 1) effectively amplified a 206 pb sequence in DNA isolated from *T. semipenetrans* juveniles extracted from citrus roots. A single sharp peak at 83.5 °C appeared upon melting curve analysis for all positive samples (Figure 1). Primer pair specificity was screened in silico using the Primer BLAST tool [22]. Sequences matching those of microorganisms and residual root cells of citrus or ‘Poncirus’ that could potentially contaminate the samples were discarded through this analysis. The amplification of similar-size products was predicted for several accessions of Nematoda and Arthropoda. However, these species are not commonly encountered on the plant and soil materials used to perform the assay, and their E values (data not presented) indicated a high probability that these matches were by chance [23]. Primer pair specificity was also tested in vitro by running qPCR in triplicate with DNA extracted from *Escherichia coli*, *C. sinensis*, and *P. trifoliata*. No positive PCR signals were detected. Samples extracted from mock-inoculated citrus roots were all PCR negative.

#### 2.1.2. Level of Detection

**Figure 2:**
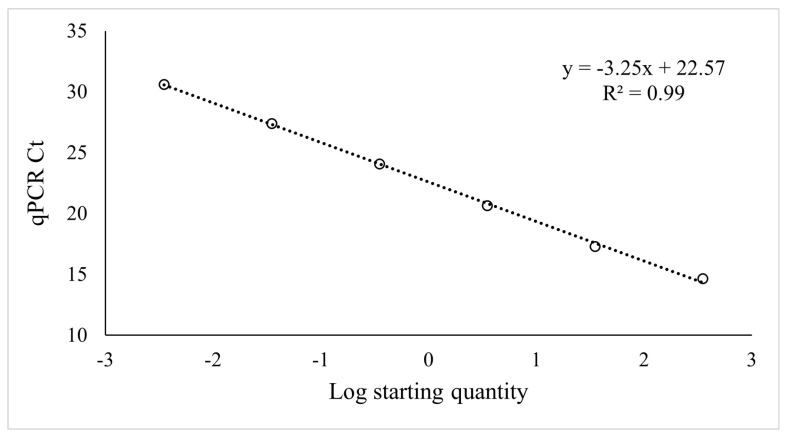
Standard curve of Ct values plotted against the log of the starting quantity of juvenile nematodes based on serial DNA preparation dilutions.

**Figure 3:**
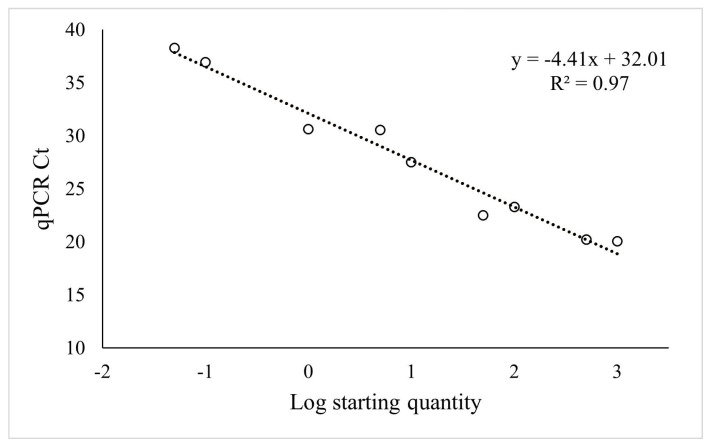
Standard curve of Ct values plotted against the log of the citrus nematode female starting quantity based on serial DNA preparation dilutions.

The primer pair was tested on serial dilutions of DNA extracted from juvenile nematodes and plotted on a log scale against Ct values. The assay detected a qPCR signal at low dilutions of DNA. A 10-fold serial dilution of 350 nematodes showed a range of Ct values from 31.4 to 14.0 (10^−5^), indicating that the assay can detect signals from the lowest level tested (0.0035 nematodes). According to the standard curve, the primers showed 103% efficiency (y = 3.25x + 22.57; R^2^ = 0.99, *p* < 0.01), which corresponds to an amplification factor of 2.0 (Figure 2). The primers were then tested on female nematode samples using a serial dilution of 1000 females. The detection limit was 0.05 nematodes, and Ct values ranged from 20.0 to 38.5. The relationship between the log concentration of nematodes and the Ct value was y = −4.41x + 32.01 (R^2^ = 0.97, *p* < 0.01; Figure 3). Thus, the assay showed 33% lower efficiency on females than on juveniles, the amplification factor for females being 1.7. For both sample types, no signal was detected in control samples, where nematodes were not added.

#### 2.1.3. Predictive Power

**Figure 4:**
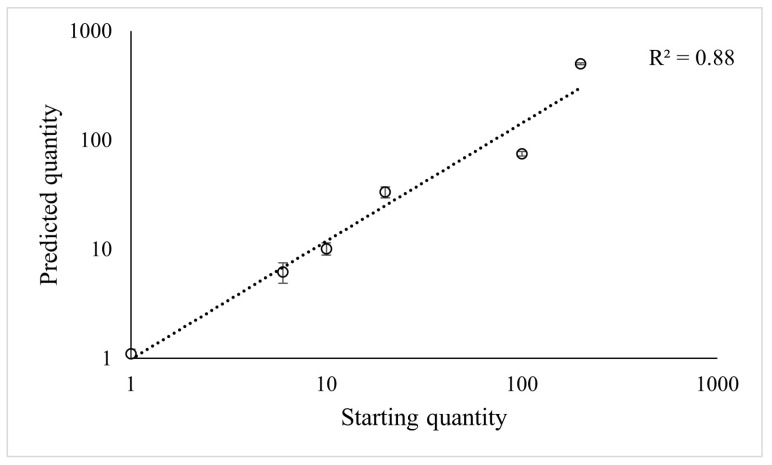
Relationship between the number of female nematodes added to the solution and the number of nematodes predicted from the Ct values using the model y = −4.41x + 32.01. Values are the means of 3 replicates and their standard error.

The relationship between the number of female nematodes added to the solution and those predicted with qPCR assay based on the model y = −4.41x + 32.01 was strong (Figure 4; F = 119.64, *p* < 0.001), and the predicted values were close to those observed with microscopic counting. However, the assay tended to underestimate the number of nematodes added to the solution at higher densities. The overall correlation between the actual number of nematodes added and those predicted with the assay was still high (R^2^ = 0.88).

### 2.2. Host Screening

**Figure 5:**
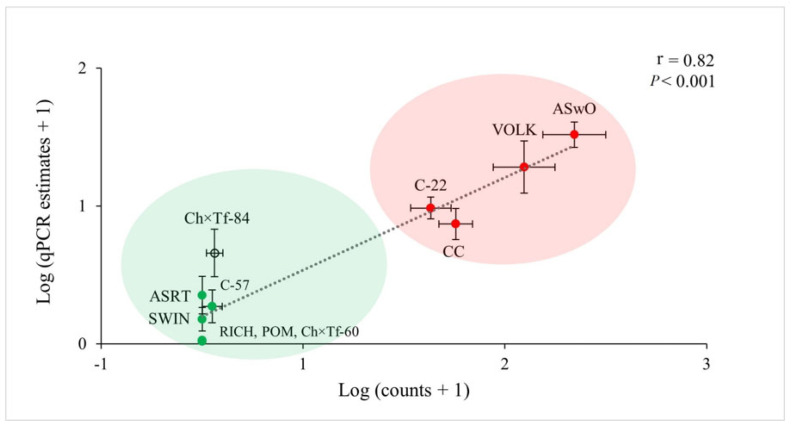
Correlation between the female nematode abundance counted on each host root and the abundance predicted from Ct values using the model y = −4.41x + 32.01. Green dots identify known resistant hosts, and red dots identify known susceptible hosts. Groups clustered within the red and green clouds were created with 2-step cluster analysis (SPSS). Values are the means of *n* = 9 and their standard error. Data are log (x + 1) transformed.

The analysis of variance performed on data from microscope counts (Table 2) showed that the females of *T. semipenetrans* varied in abundance between the 11 different rootstock hosts (*n* = 9). Resistant hosts (Rich, Pom, Swin, C-57) had a female abundance of ~0, and no significant differences were found. Hosts with unknown or uncertain susceptibility (ASRT, Ch × tf–60, and Ch × tf–84) were similar to the resistant types. Hosts known to be susceptible (CC, C-22, Volk, and ASwo) had on average about 167% more nematode females than the resistant types. Among these, Volk and ASwo had the highest female abundance, while CC and C-22 had a similar abundance that was significantly lower than that of ASwo. All the samples extracted from mock-inoculated roots (*n* = 3) were free of nematodes. Whole-root fresh weight and fibrous root fresh weight were significantly different among hosts, not correlated with female abundance (r~0) but strongly correlated to each other (r = 0.85, *p* < 0.001). The predicted female abundance from qPCR Ct values, which were calculated using the standard curve model y = −4.41x + 32.01 (Figure 3), was strongly correlated with the counted female abundance (r = 0.82, *p* < 0.001; Figure 4). Cluster analysis performed on log (x − 1)-transformed calculated and predicted female abundances identified two groups. One of the groups included all the hosts known to be susceptible, while the other group identified included all the resistant hosts and hosts with unknown suitability (Figure 5). The quality of the clustering analysis was classified as good by the statistical software.

Counted and predicted female abundances were significantly lower on hosts known to be resistant than on hosts considered susceptible (Table 3). Precisely, the counted female abundance on resistant hosts was on average 99.9% lower than on susceptible hosts, while the predicted female abundance on resistant hosts was on average 97.6% lower than on susceptible hosts. The qPCR Ct values from resistant hosts were on average 37% higher than on susceptible hosts (Table 3). Dunnett’s *t*-test was performed to compare female abundance on all the hosts tested against the standard of resistance, Rich, as previously described [21]. Comparisons based on female counts identified four hosts as significantly different from the resistance standard: CC, C-22, Volk, and ASwo. In contrast, comparisons based on female predictions identified five hosts as significantly different from the resistance standard, including the former hosts and Ch × tf–84. When Dunnett’s *t*-test was applied to Ct values, the hosts identified as susceptible coincided with the ones identified from predicted abundance data Ct (Table 3).

### 2.3. Validation in Field Samples

Root samples collected from field trees located in four different citrus-growing areas in California (Table 4) were processed for CN female extraction, DNA purification, and qPCR analysis to validate the methodology for different CN populations. The Ct values obtained for these samples were higher than 31 for rootstocks classified as resistant (C-35 and *P. trifoliata*) and lower than 29 for rootstocks classified as susceptible. Rootstocks of the *Citrus* species have, in general, lower Ct values than those of *Citrus* × *Poncirus* hybrids.

### 2.4. In-Greenhouse Rearing for Inoculum Production

**Figure 6:**
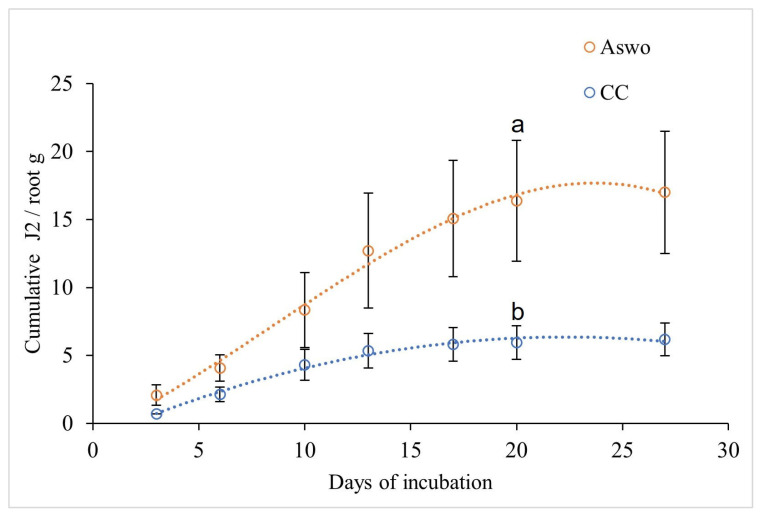
Nematode juveniles (J2) collected from the inoculum rearing on Argentina sweet orange (ASwo) and Carrizo citrange (CC) in a greenhouse after 27 days of root incubation. Data are the means of *n* = 3, bars represent the standard error, and letters indicate significant differences for the *t*-test at *p* < 0.05.

Fibrous root samples were taken from infested CC and ASwo orange plants incubated in greenhouse conditions for 28 months. Roots were collected in February and were extracted in a mist chamber for 27 days. J2 CN individuals were collected periodically and counted under a microscope. Data showed that the abundance of juveniles obtained from ASwo was about 60% higher than that from CC (Figure 6).

## 3. Discussion

For developing the new quantification methodology, we required efficient recovery of CN females from roots that yielded clean preparations suitable for microscopic counting, DNA extraction, and qPCR performance. For microscope visualization, the classical sodium-hypochlorite-acid–fuchsin staining method [25] is incompatible with qPCR analysis, but clean preparations allow counting without staining. Therefore, we modified the technique for female CN extraction from plant tissues, traditionally based on blender maceration and flotation/centrifugation [26], because it did not yield preparations of sufficient quality for in vivo microscopic counting and DNA extraction. We developed an alternative method for nematode extraction from plant tissues that is suitable for CN females and, to the best of our knowledge, has never been described in the literature before. This novel method is based on root sand abrasion and sieving. It is suitable for high-throughput sample processing, saving time and cost, yields debris-free samples that require no further centrifugation, and is suitable for in vivo microscope counting, DNA purification, and qPCR analysis.

Initially, the citrus nematode inoculum was obtained from a field at the UCR Agricultural Experiment Station, where environmental conditions limit the availability of roots containing high populations of CN females. For this reason, we used a greenhouse CN-rearing system that could yield a sufficient inoculum for performing rootstock evaluations irrespective of the season or environmental conditions. We evaluated the inoculum yield on two different susceptible hosts, Argentine sweet orange (ASwo) and Carrizo citrange (CC). Data collected indicated that ASwo yields 60% more inoculum than CC. The inoculum for host screening and for rearing initiation was obtained from *C. macrophylla* and Citremon (*C. limon* × *P. trifoliata*) roots. In this study, the inoculum showed a parasitic capability representing the CN ‘Citrus’/‘Mediterranean’ biotypes. However, future assays should address whether it is necessary to include susceptible *Citrus* × *Poncirus* hybrid hosts in the rearing to avoid possible bias in the screenings. Inoculum production in a greenhouse substantially shortened the evaluation time and saved labor costs, making the process easier and more efficient.

The selected primers successfully amplified the CN DNA, showing optimal performance on J2 preparations. However, we observed a decrease in the primer pair efficiency when testing was performed on female specimens, possibly due to the presence of inhibitors that co-purify with DNA. This issue could be further improved by adjusting the DNA extraction method. Perhaps an extraction based on magnetic beads would be more efficient at excluding inhibitors [27]. To demonstrate primer specificity in this assay, we tested non-template controls, samples from mock-inoculated seedlings, and templates from *C. sinesis*, *P. trifoliata*, and *E. coli*. We verified that the qPCR signal was only detectable when the CN DNA was present. Although non-target matching sequences were identified through the NCBI primer BLAST tool [22], these corresponded to species not expected to be found in diagnostic samples. Samples to test were CN females extracted from roots grown in a synthetic substrate pasteurized before use. Roots were thoroughly cleaned, and extracted females were sieved to 45–125 µM. Non-target matching sequences included other species from the Tylenchuloidea superfamily and insects such as *Neodiprion* spp. or other arthropods such as crabs (*Birgus* spp.). However, the E values the BLAST algorithm provided were higher in this case, indicating a lower percentage match to the primer sequences [23], potentially because of the long sequence lengths of these entries and the short lengths of the primers. If the primers do, indeed, amplify these species, potential cross-reactions could occur. We also considered using primer pairs available in the literature that are specific for *T. semipenetrans* (Table 1) [24]. However, these were unsuitable for our assay as they target an ITS region, which seems polymorphic within the species, resulting in multiple melting curves in the qPCR analysis. This limitation when using ITS regions for PCR assays has been described for other organisms, such as fungi [28]. The qPCR assay was sensitive and detected 0.0035 juveniles. This is in line with previous studies [29]. Assay sensitivity with females was lower (0.05 nematodes) but still sufficient to meet the purpose. The lower sensitivity is probably due to the presence of inhibitors. Overall, qPCR-based quantification allows for massive evaluations without relying on trained personnel or special equipment to identify and manipulate the nematode samples, making the process simpler and cost-efficient.

We tested the correlation between female abundance determined with the microscope and estimated from the qPCR Ct values using the model obtained from the CN female dilutions standard curve, y = −4.41x + 32.01, calculated from serially diluted female CN DNA (Figure 3). In our study, the female abundance determined with the microscope related well to the quantity predicted by the assay (Figure 4). This validates that abundance values predicted from Ct values can be used to assess CN resistance of the different hosts tested. We observed that qPCR tended to underestimate the abundance (Table 3), attributed to inhibitors in previous studies on other plant-parasitic species [30]. Additionally, other studies questioned whether the standard curve is more reliable when based on serial dilutions or individual samples containing different numbers of individuals [29]. In agreement with other studies [30,31,32]), we found more variation using individual samples rather than a dilution series. We also considered that the method might be more suitable and reproducible for users if the standard curve is based on DNA dilutions rather than dependent on female CN availability to prepare individual samples that require microscopic counting.

Hosts classified as resistant had a significantly lower abundance of CN females in their roots than those classified as susceptible. Those that were initially classified as unknown (Ch × Tf–84 and Ch × Tf–60) or uncertain (ASRT) were similar to resistant types (Table 2). Next, we assessed resistance by applying Dunnett’s *t*-test described by Niles et al. [21]; see Table 3. Comparing results obtained with Dunnett’s *t*-test for counted and predicted female abundance, we found a similar host classification between susceptible and resistant groups, which validates that predicted data are representative. Nevertheless, the host Ch × Tf–84 was classified as susceptible with the analysis of count data but resistant using Ct data. This discrepancy may be explained by differences in accuracy between the quantification methods. Specifically, we suspect that borderline rootstocks, such as Ch × Tf–84, may have a low abundance of females that develop slowly. This assay’s short incubation time might result in samples composed mainly of sausage-like females, which are harder to detect via visual inspection of non-stained samples than the fully developed globular females. This would create a bias affecting mainly the borderline types that the qPCR-based evaluation overcomes. In this sense, the new method should minimize ambiguous results often obtained in rootstock screenings. For instance, the rootstock ASRT was classified as ‘possibly more resistant than Troyer’ [21]. This study provides evidence to support that it can be considered resistant. For moderately sensitive and borderline types, this accurate and high-throughput quantification method offers a new opportunity to explore whether the oligogenic nature of CN resistance justifies the consideration of a tolerant category for rootstock behavior toward CN. A previous study [19] concluded that the markers developed the *Tyr1* locus were only suitable for some crossing groups and indicated some resistance in rootstocks previously classified as susceptible, such as CC. The new method could facilitate mapping studies to help characterize the genetics and physiology behind the resistance to CN and develop additional markers for improved MAS.

Further validation of these methods across a different range of samples taken from other locations and hosts would be beneficial to support the assay’s consistent performance. We provide additional data on the assay performance in samples extracted from field roots collected from different hosts and locations in California. Table 4 shows that when field samples were ranked by their Ct values, resistant hosts appeared grouped on the higher end, indicating that the qPCR assay and standards are suitable tools to discriminate host suitability to CN. These results support the fact that the new methodology developed could be used to phenotype populations in the field. It may also be helpful to assess population densities for pest management interventions or to follow up resistance durability over time in field assays.

## 4. Materials and Methods

### 4.1. Plant Materials and Growth Conditions

Fifty seeds of nine different rootstocks (Table 5) were collected from the Givaudan Citrus Variety Collection (University of California Riverside, UCR). Two individuals (84 and 60) of a Chandler pummelo (*C. maxima* (Burm.) Merr.) × Rubidoux trifoliate (*P. trifoliata*) population previously characterized as producing mostly nucellar seedlings [33] were selected from trees in field 6B located at UC Riverside’s Agricultural Experiment Station. The seeds were planted in 164 mL containers (SC10U Ray Leach by Stuewe and Sons, Tangent, OR, USA) filled with a pasteurized plaster sand and peat moss mix (9:1) and grown for 4 months in a glasshouse. Temperatures were controlled between 16 and 18 °C at night and 26 and 28 °C during the day. Relative humidity was maintained at approximately 50%. Auxiliary light was provided for 16 h daily using high-pressure sodium bulbs (Gro-Lux HPS, Sylvania, LEDVANCE North American, Wilmington, MA, USA). Pots were fertilized twice weekly (Peter’s Professional 20-20-20 by Everris International B.V.) and watered daily with 31.5 mL of water applied by drip irrigation. After 90 days, the more homogeneous seedlings (*n* = 20) of each type were screened with a set of SNP markers (Table S1) to test their nucellar origin using KASPar technology (competitive allele-specific dual Förster resonance energy transfer (FRET)-based assay for SNP genotyping). The analysis was performed by LGCgenomics, UK. Zygotic seedlings detected were discarded.

### 4.2. Nematode Inoculum Preparation from Field Trees

Fresh roots of *C. macrophylla* Wester (CRC 3842) and Citremon (*P. trifoliata* × *C. limon*) (CRC 1449) trees grafted with ‘Eureka’ lemon and located on field 18C at the UC Riverside’s Agricultural Experiment Station were collected in mid-September 2019 from soil near the irrigation lines. The roots were gently washed and divided into 12 g portions. J2 individuals were extracted from feeder roots following the mist chamber method [35] with the following modifications: the first batch of nematodes collected after 12 h of incubation was discarded, and subsequent sets were collected every 3 days for up to 27 days. Next, the extract was left to sit for 30 min before water aspiration and then rinsed twice to contain the nematode concentrate. This was resuspended in 150 mL of deionized water and stored at 6–10 °C for up to 6 days before using it for plant infestation. The inoculum was evaluated before host-screening infestations to identify the citrus nematode J2 individuals by morphological features [36] and PCR [24] using a species-specific primer set (Table 1) and to quantify viable nematodes based on body curviness and motility. This inoculum was used to inoculate the hosts for screening and to start the in-greenhouse rearing (Section 4.4).

### 4.3. Host Infestation and Incubation

For each rootstock tested, nine 4-month-old seedlings were inoculated by applying 10 mL of field inoculum suspension containing 45–350 viable J2 nematodes/mL weekly for 4 weeks. Three similar plants of each variety were mock-inoculated with a root extract prepared from non-infested *C. macrophylla* roots and sieved to 25 µm. Irrigation was controlled manually for 5 days after inoculation to avoid inoculum draining. Fertilization was not applied during the inoculation weeks. Plants were randomly distributed in the greenhouse benches and incubated for 4 months after inoculation under the described growth conditions and were periodically pruned and treated for aerial pests, as needed. Seedlings were then uprooted; the soil was carefully removed manually to process the roots for female nematode extraction.

### 4.4. In-Greenhouse Rearing for Inoculum Production

Carrizo citrange (*C. sinensis* × *P. trifoliata*) and Argentina sweet orange that had been infested and incubated, as described in Section 4.3, were transplanted to 3 L pots and kept for 24 months. Fibrous roots were then collected and used for inoculum preparation, as described. Three replicates of each host were incubated, and J2 CN individuals were extracted and counted under the microscope (S8APO, Leica, Wetzlar, Germany) every 3 days for 27 days. The greenhouse inoculum source was helpful for future experiments.

### 4.5. Female Nematode Extraction

Root systems were detached from the plant and gently washed with tap water; excess water was carefully wiped with a paper towel. Fibrous roots were separated from the tap root, weighted, chopped into 1 cm sections, and placed into 50 mL conical centrifuge tubes (Corning, NY, USA). Samples heavier than 6 g were split into 2 tubes. They were filled with a 1.5% NaClO solution (Clorox, Oakland, CA, USA), incubated for 4 min, and rinsed with tap water. The tubes were then filled with deionized water (Merck, Millipore, Burlington, MA, USA), incubated for 15 min on a rotating mixer (Loopster Basic, IKA, Wilmington, NC, USA), and rinsed twice with deionized water to eliminate CN eggs. Roots were carefully tapped dry with a paper towel, air-dried overnight at 4 °C, and placed back into the 50 mL conical centrifuge tubes containing 10 mL of silica sand (Sigma-Aldrich, San Luis, MO, USA; previously washed, sieved to a 500–150-µm size, autoclaved for 10 min at 121 °C, and air-dried). To cut off the posterior end of the CN females from the roots, each tube was vortexed twice for 5 min at 3000 RPM on a digital vortex (Genie^®^ 2 Timed Mixer) using the Genie^®^ 504-0039-00 platform. A 10 s manual agitation step was performed between each 5 min vortex. Samples were then sieved through 500, 125, and 45 µm sieves (Cole-Parmer, Vernon Hills, IL, USA). The 45 µm sieve was carefully rinsed with deionized water. The posterior ends of the female nematodes were collected in a 15 mL conical centrifuge tube (Corning, NY, USA). They were concentrated into a 2 mL solution by letting them settle overnight and carefully removing the supernatant water volume via aspiration.

### 4.6. Nematode Quantification

#### 4.6.1. Based on Microscopy

Solutions containing J2 CN individuals were vortexed for 5 s, and 500 µL of the suspension was loaded onto each chamber of a Mc-Master slide (Chalex Corp, Portland, OR, USA). Nematodes were counted in triplicate with a S8APO microscope at 4× zoom.

#### 4.6.2. Based on Real-Time PCR

##### DNA Extraction

For testing primer efficiency, DNA was extracted from J2 CN individuals following the lysis method [37]. For screening experiments and standard curves, solutions containing a known number of posterior ends of CN females were placed in bead-beating tubes and extracted using a DNeasy Plant Pro Kit (Qiagen, Hilden, Germany), as specified by the most recent available manufacturer instructions (August 2019), with the following modifications: 50 µL of solution PS was added together with solution CD1, and homogenization was performed at 6.0 m/s for 180 s on a Fast-Prep-24 bead beater (MP Biomedicals, CA, USA), followed by 90 min digestion at 55 °C with 16 U of proteinase K (New England Biolabs Inc., Beverly, MA, USA). A subsequential 60 s bead-beating step was performed before the first centrifugation step (12,000× *g* for 2 min). DNA was eluted in 100 µL of nuclease-free water. Samples from the host-screening experiment were diluted 1:10. DNA quality and concentration were assessed in an ND-1000 full spectrum UV–Vis spectrophotometer (Nanodrop Technologies, Wilmington, DE, USA).

##### qPCR Assay Design and Validation

The primer set TS18SAJ966511.1 (Table 1) was developed using open-source Primer3 software [38] to target the 18S rRNA gene of *T. semipenetrans* (AJ966511) retrieved from GenBank (NCBI). DNA was extracted from solutions with a known number of J2 CN individuals and serially diluted to determine primer efficiency, assay specificity, and the limit of detection. Primers were then tested on serial dilutions of DNA extracted from CN females’ posterior ends and DNA solutions from a known number of CN females’ posterior ends. Three technical replicates of qPCR were performed for each concentration and averaged. The qPCR assays were performed on a CFX96 Touch Real-Time PCR Detection System (Bio-Rad Laboratories, Inc., Hercules, CA, USA), and data were analyzed using Bio-Rad CFX Manager software (v3.1). The reactions were performed in a total volume of 20 µL using Sso Advanced Universal SYBR Green Supermix (Bio-Rad) with 2.0 µL of a DNA template for screening experiments, 1.0 µL of a DNA template for standard curves, and 10 µM primer concentration. The cycling conditions were 98 °C for 5 min, 39 cycles of 98 °C for 15 s, and 63 °C for 40 s. Non-template controls were loaded with DNAase-free water instead of the template. Ct values were determined using the program’s default settings. Reactions were run in 3 technical triplicates for each sample and averaged. The primer efficiency was calculated as E = −1 + 10^(−1/slope), where 10^(−1/slope) is the amplification factor.

#### 4.6.3. Modeling

To generate standard curves, female nematode numbers in dilution samples (1000, 100, 10, 5, 1, 0.1, 0.05) were log-transformed and plotted against the Ct values obtained. Predictions of female abundance were made from the experimental qPCR Ct values using the linear model equation that describes the standard curve. Next, samples containing a known number of posterior ends of CN females (1, 6, 10, 20, 100, 200) were tested with qPCR. Linear regression analysis was performed to determine to what degree data predicted from qPCR signals related to female nematode counts determined using microscopic evaluation.

### 4.7. Statistical Analysis

Experimental data on female abundance obtained from the host screening were log (x − 1)-transformed to meet normality and homoscedasticity, as described in Niles et al. [21]. An analysis of variance was performed on the abundance of citrus nematode females per root and the root fresh weight for the different hosts screened. The correlation between transformed predictions and counts was tested with Pearson’s coefficient (r), and data were grouped using two-step cluster analysis. Predicted and counted transformed abundances and Ct values were then analyzed using ANOVA to compare groups with different host suitability. Each host was compared against the standard for resistance using bilateral Dunnett’s *t*-test. All analyses were performed in the statistical program IBM SPSS Statistics for Windows, version 28.0. (IBM Corp. Released 2021, Armonk, NY, USA).

### 4.8. Validation of Field Samples

Fibrous roots from field trees were collected manually from the locations and hosts specified in Table 4. Posterior ends of CN females were extracted from 4 g of fibrous roots using the method described. Their DNA was purified and quantified using qPCR, as described.

## 5. Conclusions

This new methodology allows extraction of CN females and quantification of their abundance in roots more efficiently than the previously standard staining technique. The novel nematode extraction method from roots yields extracts suitable for molecular lab work. The nematode quantification approach based on real-time PCR provides a more systematic, reproducible, and accurate way to screen for CN resistance. This study also contributes to the growing literature that indicates it is possible to quantify nematodes using molecular diagnosis. As pest management options become more restrictive, the need for resistant plants and affordable nematode testing is expected to increase. This method can save time and costs, allowing to scale the nematode testing to high-throughput workflows.

## Figures and Tables

**Table 1:** Real-time PCR (qPCR) primers used to identify and quantify the citrus nematode.

Primer Name	Sequences (5′→3′)	Target	Reference
TS18SAJ966511.1-228F	ATCGTATGGGCTTGTCCCGA	18s rRNA	This study
TS18SAJ966511.1-433R	TCGTCACTACCTCCTCGTGC		
TW81	GTTTCCGTAGGTGAACCTGC	ITS rRNA	[24]
Semipenetrans specific	GGACTCTGCTCAACCTGGTAGA		

**Table 2:** Analysis of variance of the abundance of citrus nematode females per root counted and the root fresh weight for the different hosts tested.

Host	^1^ Females/Root	Whole-Root FW (g)	Fibrous Root FW (g)
Rich—^2^ R	0.00 a	9.38 ab	4.07 a
Pom—R	0.00 a	12.03 a	5.87 ab
Ch × tf–60—U	0.00 a	17.04 bc	7.44 bc
Swin—R	0.00 a	19.46 c	11.66 d
ASRT—U	0.00 a	14.05 abc	7.36 bc
C-57—R	0.29 a	25.20 d	11.74 d
Ch × tf–84—U	0.46 a	16.82 bc	8.68 c
CC—S	84.55 bc	17.53 bc	8.72 c
C-22—S	61.93 b	13.85 abc	7.31 bc
Volk—S	189.59 cd	15.01 abc	6.20 abc
ASwo—S	341.33 d	17.26 bc	8.56 bc
Average	5.35	16.15	7.96
ANOVA	F = 136.26, *p* < 0.001	F = 4.33, *p* < 0.001	F = 7.00, *p* < 0.001

^1^ Analyses were performed on log-transformed (log [x + 1]) female nematode abundance; data presented are the back-transformed means of *n* = 9. ^2^ Prior evidence of host susceptibility is indicated as R, resistant; S, susceptible; and U, unknown or uncertain. Letters indicate significant differences at *p* < 0.05.

**Table 3:** Comparison of the analysis of variance of the abundance of females counted, the abundance of females predicted based on Ct values, and the qPCR Ct values for each host tested.

Host	^1,2^ Females Counted	^1,3^ Females Predicted with Ct Values	qPCR Ct Value
Rich—^4^ R	0.00	0.04	38.3
Pom—R	0.00	0.05	38.4
Ch × tf–60—U	0.00	0.06	38.1
Swin—R	0.00	0.51	35.9
ASRT—U	0.12	0.87	34.5
C-57—R	0.00	1.26	34.0
Ch × tf–84—U	0.15	3.57 ***	31.2 ***
CC—S	17.06 ***	6.39 ***	28.7 ***
C-22—S	12.59 ***	8.65 ***	28.0 ***
Volk—S	38.51 ***	18.15 ***	26.7 ***
ASwo—S	68.89 ***	31.86 ***	25.5 ***
Average R vs. S	0.03 vs. 27.69	0.33 vs. 13.56	37.36 vs. 27.22
ANOVA	F = 343.65, *p* < 0.001	F = 163.58, *p* < 0.001	F = 66.80, *p* < 0.001
Dunnett’s		3.050 _98, 0.001_	

^1^ Analyses were performed on log-transformed (log [x + 1]) nematode abundances; data presented are the back-transformed means of *n* = 9. ^2^ Counts per root × 0.2 (dilution factor). ^3^ Prediction based on the standard curve y = −4.41x + 32.01. ^4^ Prior evidence of host susceptibility is indicated as R, resistant; S, susceptible; and U, unknown or uncertain. *** Indicates that it is significantly higher than the resistant standard for Dunnett’s *t*-test at *p* < 0.001.

**Table 4:** Field trees’ root samples tested using qPCR for abundance of citrus nematode females.

Rootstock	Pedigree	^1^ Ct value	^2^ Sampling Location
Koethen sweet orange	*C. sinensis* L. Osbeck	24.0	LR
Rangpur lime	*C. limonia*	24.2	SCREC
Alemow	*C. macrophylla* W	25.2	LR
Volkamer lemon	*C. volkameriana*	25.7	CVARS
Alemow	*C. macrophylla* W	26.1	LR
Yuma ponderosa	*C. maxima* ×?	26.4	SCREC
Troyer citrange	*C. sinensis* × *P. trifoliata*	26.4	LREC
Schaub rough lemon	*C. jambhiri* Lush	27.5	LR
C-32	*C. sinensis* × *P. trifoliata*	28.3	LR
Carrizo citrange	*C. sinensis* × *P. trifoliata*	28.4	SCREC
Tosu hybrid	*Citrus* × *aurantium* L.	28.4	LR
Carrizo citrange	*C. sinensis* × *P. trifoliata*	28.5	CVARS
C-32	*C. sinensis* × *P. trifoliata*	28.9	SCREC
C-35	*C. sinensis* × *P. trifoliata*	31.2	CVARS
C-35	*C. sinensis* × *P. trifoliata*	31.7	LREC
Flying dragon	*P. trifoliata* L. Raf	32.0	SCREC

^1^ Cycle threshold values are the means of 3 technical replicates. ^2^ LREC (36 21′26″ N, 119 03′19″ W; Lindcove Research and Extension Center, Exeter, CA, USA; 155 m of altitude); CVARS (33 31′16″ N, 116 09′17″ W; Coachella Valley Agricultural Research Station, Thermal, CA, USA; 5 m of altitude); LR (34 18′09″ N, 119 07′15″ W; Limoneira Lower 28 Ranch, Santa Paula, CA, USA; 54 m of altitude); SCREC (33 41′32″ N, 117 43′18″ W; South Coast Research and Extension Center, Irvine, CA, USA; 124 m of altitude).

**Table 5:** Rootstocks used as hosts for screening, abbreviations, and suitability.

Rootstock	Species/Pedigree	Abbreviation	^1^ Host Suitability	References
Rich 16-6 trifoliate	*Poncirus trifoliata* (L.) Raf	Rich	R	[19]
Pomeroy trifoliate		Pom	R	[19,34]
Swingle citrumelo	*Citrus paradisi* × *P. trifoliata* Macfad.	Swin	R	[19,21,34]
Furr C-57 citrandarin	*C. sunki* (Hayata) hort ex. Tanaka × *P. trifoliata* ‘Swingle’	C-57	R	[21]
African shaddock × Rubidoux trifoliate	*C. maxima* Merr. × *P. trifoliata*	ASRT	U	[21]
Carrizo citrange	*C. sinensis* Osb. × *P. trifoliata*	CC	S	[19,21,34]
Bitters C-22 citrandarin	*C. sunki* × *P. trifoliata* ‘Swingle’	C-22	S	[21]
Volkamer lemon	*C. limonia* Osb.	Volk	S	[19]
Argentina sweet orange	*C. sinensis* Osb.	ASwo	S	[19,21,34]
Chandler × trifoliate–84	*C. maxima* Merr × *P. trifoliata*	Ch × tf–84	U	[33]
Chandler × trifoliate–60		Ch × tf–60	U	[33]

^1^ R, resistant; S, susceptible; U, unknown/uncertain.

## Data Availability

The data presented in this study are available on request from the corresponding author.

## Supplementary Materials

The following supporting information can be downloaded at https://www.mdpi.com/article/10.3390/plants12132543/s1: Table S1: KASP markers analyzed in host seedlings to verify they were clonal.

## References

[1] Duncan L.W., Ciancio A., Mukerji K. (2009). Managing Nematodes In Citrus Orchards. Integrated Management of Fruit Crops Nematodes.

[2] Abd-Elgawad M.M., Koura F.F., Montasser S.A., Hammam M.M. (2016). Distribution and losses of *Tylenchulus semipenetrans* in citrus orchards on reclaimed land in Egypt. Nematology.

[3] Shokoohi E., Duncan L.W., Sikora R., Coyne D., Hallmann J., Timper P. (2018). Nematodes parasites of citrus. Plant Parasitic Nematodes in Subtropical and Tropical Agriculture.

[4] Duncan L.W., Timmer L.W., Duncan L.W. (1999). Nematode diseases of citrus. Citrus Health Management.

[5] Sorribas F.J., Verdejo-Lucas S., Pastor J., Ornat C., Pons J., Valero J. (2008). Population densities of *Tylenchulus semipenetrans* related to physicochemical properties of soil and yield of clementine mandarin in Spain. Plant Dis..

[6] Liu G., Chen J., Xiao S., Zhang S.S., Pan D.M. (2011). Development of Species-Specific PCR Primers and Sensitive Detection of the *Tylenchulus semipenetrans* in China. Agric. Sci. China.

[7] Song Z.Q., Cheng J.E., Cheng F.X., Zhang D.Y., Liu Y. (2017). Development and Evaluation of Loop-Mediated Isothermal Amplification Assay for Rapid Detection of *Tylenchulus semipenetrans* Using DNA Extracted from Soil. Plant Pathol. J..

[8] Van Gundy S.D. (1958). The life history of the citrus nematode, *Tylenchulus semipenetrans*. Nematologica.

[9] Grafton-Cardwell E.E., Baldwin R.A., Becker J.O., Eskalen A., Lovatt C.J., Rios S., Adaskaveg J.E., Faber B.A., Haviland D.R., Hembree K.J. (2021). UC IPM Pest Management Guidelines: Citrus.

[10] Kaplan D.T. (1981). Characterization of citrus rootstock responses to *Tylenchulus semipenetrans* (Cobb). J. Nematol..

[11] Van Gundy S.D., Kirkpatrick J.D. (1964). Nature of resistance in certain citrus root-stocks to citrus nematode. Phytopathology.

[12] Sato K., Kadota Y., Shirasu K. (2019). Plant Immune Responses to Parasitic Nematodes. Front. Plant Sci..

[13] Verdejo-Lucas S., Sorribas F.J., Pons J., Forner J.B., Alcaide A. (1997). The Mediterranean biotypes of *Tylenchulus semipenetrans* in Spanish citrus orchards. Fundam. Appl. Nematol..

[14] Baines R.C., Cameron W., Soost R.K. (1974). Four biotypes of *Tylenchulus semipenetrans* in California identified, and their importance in the development of resistant citrus rootstocks. J. Nematol..

[15] Gottlieb Y., Cohn E., Spiegel-Roy P. (1986). Biotypes of the citrus nematode (*Tylenchulus semipenetrans* Cobb) in Israel. Phytoparasitica.

[16] Murguía C., Abad P., Jordá C., Bello A. (2005). Identification of the Poncirus biotype of *Tylenchulus semipenetrans* in Valencia, Spain. Span. J. Agric. Res..

[17] Kaplan D.T., Starr J.L. (1990). Screening for resistance to *Tylenchulus semipenetrans* and *Radopholus* species. Methods for Evaluating Plant Species for Resistance to Plant Parasitic Nematodes.

[18] Ling P., Duncan L.W., Deng Z., Dunn D., Hu X., Huang S., Gmitter F.G. (2000). Inheritance of citrus nematode resistance and its linkage with molecular markers. Theor. Appl. Genet..

[19] Xiang X., Deng Z., Chen C., Gmitter F., Bowman K. (2010). Marker Assisted Selection in Citrus Rootstock Breeding Based on a Major Gene Locus ‘Tyr1’ Controlling Citrus Nematode Resistance. Agric. Sci. China.

[20] Xu X., Zhan’ao D., Qifa Z., Cunxian C., Gmitter F.G. (2009). Developing specific markers and improving genetic mapping for a major locus *Tyr1* of Citrus nematode resistance. Mol. Plant Breed..

[21] Niles R.K., Freckman D.W., Roose M.L. (1995). Use of trifoliate orange as a comparative standard for assessing the resistance of citrus rootstocks to citrus nematode. Plant Dis..

[22] Ye J., Coulouris G., Zaretskaya I., Cutcutache I., Rozen S., Madden T. (2012). Primer-BLAST: A tool to design target-specific primers for polymerase chain reaction. BMC Bioinform..

[23] Altschul S.F., Gish W. (1996). Local alignment statistics. Methods Enzymol..

[24] Maafi T.Z., Amani M., Stanley J.D., Inserra R.N., Van den Berg E., Subbotin S.A. (2012). Description of *Tylenchulus musicola* sp. n. (Nematoda: Tylenchulidae) from banana in Iran with molecular phylogeny and characterisation of species of *Tylenchulus* Cobb, 1913. Nematology.

[25] Byrd D.W., Kirkpatrick T., Barker K.R. (1983). An improved technique for clearing and staining plant tissue for detection of nematodes. J. Nematol..

[26] EPPO (2013). Nematode extraction PM 7/119 (1). EPPO Bull..

[27] Berensmeier S. (2006). Magnetic particles for the separation and purification of nucleic acids. Appl. Microbiol. Biotechnol..

[28] Woo P.C., Leung S.Y., To K.K., Chan J.F., Ngan A.H., Cheng V.C., Lau S.K., Yuen K.Y. (2010). Internal transcribed spacer region sequence heterogeneity in *Rhizopus microsporus*: Implications for molecular diagnosis in clinical microbiology laboratories. J. Clin. Microbiol..

[29] Hodson A.K., Cicchetto A., Fierro F.A. (2021). Real time PCR assays to detect and quantify the nematodes *Pratylenchus vulnus* and *Mesocriconema xenoplax*. Crop Prot..

[30] Yan G., Smiley R.W., Okubara P.A. (2012). Detection and quantification of Pratylenchus thornei in DNA extracted from soil using real-time PCR. Phytopathology.

[31] Min Y.Y., Toyota K., Sato E. (2012). A novel nematode diagnostic method using the direct quantification of major plant-parasitic nematodes in soil by real-time PCR. Nematology.

[32] Sato E., Min Y.Y., Shirakashi T., Wada S., Toyota K. (2007). Detection of the root-lesion nematode, *Pratylenchus penetrans* (Cobb), in a nematode community using real-time PCR. Jpn. J. Nematol..

[33] Kepiro J.L., Roose M.L. (2010). AFLP markers closely linked to a major gene essential for nucellar embryony (apomixis) in *Citrus maxima* × *Poncirus trifoliata*. Tree Genet. Genomes.

[34] McCarty C.D., Bitters W.P., Van Gundy S.D. (1979). Susceptibility of 25 Citrus Rootstocks to the Citrus Nematode. HortScience.

[35] Ravichandra N.G. (2014). Nematological techniques. Horticultural Nematology.

[36] Mai W.F., Mullin P.G., Lyon H.H., Loeffler K. (1996). Plant-Parasitic Nematodes: A Pictorial Key to Genera.

[37] Braun-Kiewnick A., Viaene N., Folcher L., Ollivier F., Anthoine G., Niere B., Sapp M., van de Vossenberg B., Toktay H., Kiewnick S. (2016). Assessment of a new qPCR tool for the detection and identification of the root-knot nematode *Meloidogyne enterolobii* by an international test performance study. Eur. J. Plant Pathol..

[38] Untergasser A., Cutcutache I., Koressaar T., Ye J., Faircloth B.C., Remm M., Rozen S.G. (2012). Primer3 new capabilities and interfaces. Nucleic Acids Res..

