# Supplementary material for: Real-Time PCR to Phenotype Resistance to the Citrus Nematode Tylenchulus semipenetrans Cobb
Source: Plants (Basel). 2023 Jul 4;12(13):2543. doi: 10.3390/plants12132543 (PMC10346647; doi:10.3390/plants12132543)
Supplement: Supplementary file 1 [file plants-12-02543-s001.zip › plants-2452583-SI.pdf]

plants MDPI

Table S1. KASP markers analyzed in host seedlings to verify they were clonal.

Name	Location (Chromosome/Pair Base)
AX-160721997	5/41365556
AX-159972794	4/24346578
AX-160091999	1/27769136
AX-160240249	2/11394067
AX-160809884	8/21061871
AX-160076939	1/27016361
AX-160106432	6/25358893
AX-160696595	9/30529455
AX-161079647	3/50656481
AX-160311246	5/40528286
AX-160992153	7/783299
AX-160797457	1/21345422

Location is the SNP location in the *Citrus clementina* v1.0 genome sequence.
